# Heterojunction Derived Efficient Charge Separation for High Sensitivity Self‐Powered Flexible Photodetectors toward Real‐Time Heart Rate Monitoring

**DOI:** 10.1002/advs.202505945

**Published:** 2025-05-24

**Authors:** Nan Ding, Ge Zhu, Xiaotao Zhang, Wen Xu, Hailong Liu, Yanan Ji, Yuanzheng Chen, Bin Dong

**Affiliations:** ^1^ Key Laboratory of New Energy and Rare Earth Resource Utilization of State Ethnic Affairs Commission School of Physics and Materials Engineering Dalian Minzu University Dalian 116600 China; ^2^ School of Physical Science and Technology Southwest Jiaotong University Chengdu 610031 China; ^3^ Zhuhai Beijing Institute of Technology (BIT) Beijing Institute of Technology Zhuhai 519088 China

**Keywords:** charge separation, flexible self‐powered photodetectors, heart rate monitoring, heterojunctions, perovskite quantum dots

## Abstract

Real‐time and accurate heart rate monitoring is crucial in the field of disease prevention and early diagnosis. Compared with the conventional rigid heart rate sensors, wearable flexible devices have unique advantages, such as convenient, high comfortable to the skin, and low data extraction errors. Currently, the available flexible electronic devices encounter with large power consumption, low detectivity, and slow response time, restricting their further commercial applications. Herein, flexible self‐powered photodetectors (PDs) are developed by the synergistic strategy of engineering CsPbI_3_:Ho^3+^@SnS quantum dots (QDs) *p*‐*n* heterojunctions and doping SnS QDs into spiro‐OMeTAD hole transport layer (HTL). The designing CsPbI_3_:Ho^3+^@SnS QDs *p*‐*n* heterojunctions as the photosensitive layer to effectively enhance the built‐in field, reduce defect density, and boost the charge separation efficiency. Meanwhile, the high hole mobility and suitable energy band structure of *p*‐type SnS QDs are doped into spiro‐OMeTAD HTL, which can improve the hole extraction, and balance electron and hole mobilities. Such flexible self‐powered PDs exhibit excellent sensitivity and stability with high responsivity (0.58 A W^−1^) and detectivity (1.13×10^13^ Jones), and fast response time (98.8 µs). The flexible self‐powered PDs are further integrated with the light‐emitting diodes (LEDs) as a photoplethysmography (PPG) system, realizing real‐time and accurate heart rate monitoring.

## Graphical abstract

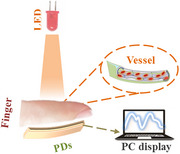

## 1 Introduction

Real‐time and accurate monitoring of life signals is a key component of health care, especially for those people with serious cardiovascular disease and elderly patients with mobility problems. Heart rate is one of the most valuable tracked vital signs, which can be obtained by electrocardiogram,^[^ ^1^ ^]^ photoplethysmography (PPG),^[^ ^2^ ^]^ thoracic motion tracking,^[^ ^3^ ^]^ and ballistocardiography.^[^ ^4^ ^]^ Among them, commercial PPG devices composed of light‐emitting diodes (LEDs) and Si photodetectors (PDs), have become an ideal technique for heart rate monitoring, due to noninvasive, hygienic, and convenient.^[^ ^5^, ^6^ ^]^ However, in our daily life, the human body is constantly moving, and the uncomfortable to the skin, poor data extraction precision, and rigidity for Si PDs hinder the PPG device's further development. Wearable flexible electronic devices exhibit unprecedented mechanical compliance and skin compatibility, which can achieve accurate heart rate monitoring, even under dynamic working conditions.^[^ ^7^, ^8^ ^]^ Organic semiconductors have emerged as promising optoelectronic materials for flexible electronic devices, due to their high absorption coefficient, low‐cost manufacturing, and compatibility with lightweight and flexible substrates.^[^ ^9^, ^10^ ^]^ However, the low carrier mobility, inefficient charge extraction, and the disordered molecular alignment of organic molecules result in large power consumption, low detectivity, and limited response time. Thus, developing high sensitivity and fast response time flexible self‐powered PDs for wearable PPG systems is in urgent demand.

Colloidal CsPbI_3_ perovskite quantum dots (PQDs) with effective charge mobility, long carrier diffusion length, and tunable bandgaps, making them excellent photoresponse layer material for PDs.^[^ ^11^, ^12^, ^13^ ^]^ Meanwhile, CsPbI_3_ PQDs show an enormous advantage for flexible optoelectronic devices, owing to its fabrication without high‐temperature annealing. Currently, the construction of *p*‐*n* heterojunctions has become a promising way to realize self‐powered PDs, enabling high responsivity and fast response time.^[^ ^14^, ^15^ ^]^ Nevertheless, the low electron‐hole separation efficiency, poor stability, and high trap density (uncoordinated Pb^2+^ and halide vacancies) of CsPbI_3_ PQDs prevent the applications for *p*‐*n* heterojunctions self‐powered PDs.^[^ ^16^, ^17^ ^]^ Moreover, the unbalanced interface charge transport properties between the perovskite layer and electronic transport layer (ETL) or hole transport layer (HTL), and the mismatch heterojunction energy levels, which can cause the hole accumulation at perovskite layer/ETL interface, deteriorating the performances of PDs.^[^ ^18^, ^19^ ^]^

Binary sulfide, including MoS_2_, Cu*_x_* S, SnS, PbS, and Sb_2_S_3_, as typical of *p*‐type semiconductors shows excellent hole mobility and stability, making them promising materials for constructing *p*‐*n* heterojunctions self‐powered PDs.^[^ ^20^, ^21^, ^22^ ^]^ Particularly, SnS quantum dots (QDs) could be the best *p*‐type semiconductor choice, because of its suitable optical bandgap (≈1.3 eV), high carrier concentration (≈10^14^–10^17^ cm^−3^), and chemical stability.^[^ ^23^, ^24^, ^25^ ^]^ Meanwhile, the SnS QDs with electron‐rich surface functional groups can also serve as the Lewis base additive to form strong coordination bonds with the uncoordinated Pb^2+^, reducing the trap density of perovskite.^[^ ^26^, ^27^ ^]^ Besides, the SnS QDs with suitable energy band structure and high hole mobility (≈90 cm^2^ V^−1^ s^−1^) would provide a “bridge” between perovskite layers and HTL, enhancing the hole extraction and transport for PDs.^[^ ^24^ ^]^

Herein, the CsPbI_3_:Ho^3+^@SnS QDs *p*‐*n* heterojunction can be constructed by an electrostatic self‐assembly method, as the photoresponsivity layer for self‐powered PDs. Meanwhile, the introduction of SnS QDs into spiro‐OMeTAD (Spiro+SnS) forms a composite HTL, exhibiting efficient charge extraction and transportation, and better‐matching energy level between the photoresponsivity layer and metal electrode compared with the pristine spiro‐OMeTAD (Spiro) HTL. The self‐powered PDs based on the FTO/SnO_2_/CsPbI_3_:Ho^3+^@SnS QDs *p*‐*n* heterojunction/Spiro+SnS/Au show a responsivity of 0.76 A W^−1^ and detectivity of 1.51×10^13^ Jones. Furthermore, the high detectivity (1.13×10^13^ Jones) and mechanical stability of the flexible self‐powered PDs can be obtained with polyethylene terephthalate (PET) substrate, achieving accurate and real‐time heart rate monitoring by the PPG system.

## 2 Results and Discussion

**Figure 1:**
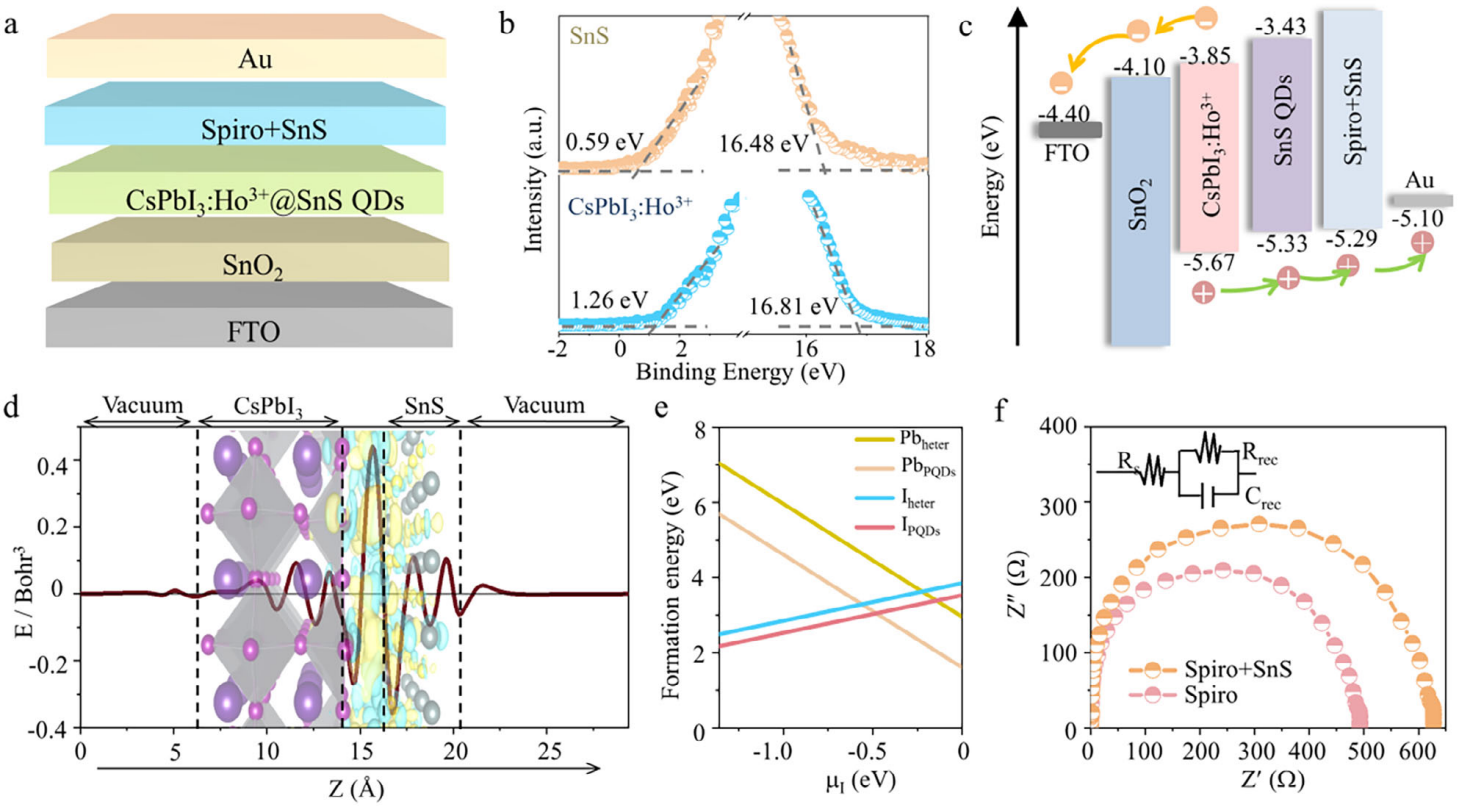
a) Schematic image of the self‐powered PDs. b) UPS spectra of SnS QDs and CsPbI_3_:Ho^3+^ PQDs. c) Energy level alignment of the self‐powered PDs. d) Charge density difference and the average charge difference along the z‐direction of CsPbI_3_:Ho^3+^@SnS heterojunctions. The yellow and blue regions indicate the charge density accumulation and depletion. The surface value is set to 0.0005e Bohr^−3^. e) The I and Pb defect formation energies for CsPbI_3_:Ho^3+^ and CsPbI_3_:Ho^3+^@SnS heterojunctions with respect to the chemical potential of the I atom. f) Nyquist plots of Spiro and Spiro+SnS films at 0 V under the dark. The inset shows the equivalent circuit for the impedance spectroscopy.

**Figure** **1**a shows the self‐powered PDs with the structure of FTO/SnO_2_/CsPbI_3_:Ho^3+^@SnS QDs heterojunctions/SnS QDs doped spiro‐OMeTAD (Spiro+SnS)/Au from the bottom to the top layer. The Fermi level of SnS QDs and CsPbI_3_:Ho^3+^ PQDs were calculated to be −4.74 and −4.41 eV, respectively, based on ultraviolet photoelectron spectroscopy (UPS) measurements (Figure 1b), suggesting the *p*‐type and *n*‐type of SnS QDs and CsPbI_3_:Ho^3+^ PQDs. According to the bandgaps of SnS QDs (1.9 eV) and CsPbI_3_:Ho^3+^ PQDs (1.82 eV) in Figure S1 (Supporting Information), the valence band maximum (VBM) and conduction band minimum (CBM) of SnS QDs and CsPbI_3_:Ho^3+^ PQDs are −5.33 and −5.67 eV, −3.63 and −3.93 eV, respectively. As illustrated in Figure 1c, the band alignment between CsPbI_3_:Ho^3+^ PQDs and SnS QDs enables them to form a type‐II *p*‐*n* heterojunction. Under light illumination, the generated electron in CsPbI_3_:Ho^3+^ PQDs tends to ETL (SnO_2_), while the hole moves to the SnS QDs layer, subsequently transferring to HTL (Spiro+SnS).

To explore the optical properties of the interface bonding, charge density difference, and Bader charge analysis were obtained to examine the charge transfer of interfacial atoms by density functional theory (DFT) calculations (Figure 1d; Note S1, Supporting Information). The electrons accumulate and the charge density increases at the interface of SnS/CsPbI_3_:Ho^3+^, which is crucial for enhancing photoelectric performance.^[^ ^28^, ^29^ ^]^ Furthermore, the electrons flow from SnS to CsPbI_3_:Ho^3+^ due to the depletion of charge density in the region of SnS QDs. The combination between SnS QDs and CsPbI_3_:Ho^3+^ PQDs can be the form of a type‐II *p*‐*n* heterojunction in Figure S2 (Supporting Information), aligning with the experimental observation. Moreover, we assessed the defect formation energies of I vacancies and Pb vacancies in CsPbI_3_:Ho^3+^ PQDs (I_PQDs_ and Pb_PQDs_) and CsPbI_3_:Ho^3+^@SnS QDs type‐II *p*‐*n* heterojunctions (I_heter_ and Pb_heter_) under the different component circumstances. It can be observed that within the range of I atom potential (μ_I_) changes, both I_heter_ and Pb_heter_ exhibit higher defect formation energies than that of I_PQDs_ and Pb_PQDs_ (Figure 1e), suggesting that the defects of CsPbI_3_:Ho^3+^ PQDs were effectively passivated after combined with SnS QDs.^[^ ^30^, ^31^ ^]^ To further understand the whole dynamic of the Spiro layer with and without the SnS QDs doping, the Nyquist plots of Spiro and Spiro+SnS were measured in Figure 1f. The larger recombination resistance (*R_rec_*) was obtained in Spiro+SnS film (618.9 Ω) compared to the only Spiro layer (495.7 Ω), indicating the charge recombination can be effectively suppressed.^[^ ^32^, ^33^ ^]^ Based on those results, the synergistic engineering of building CsPbI_3_:Ho^3+^@SnS QDs type‐II *p*‐*n* heterojunctions and doping SnS QDs into Spiro layer are favorable for realizing the highly sensitive self‐powered PDs.

**Figure 2:**
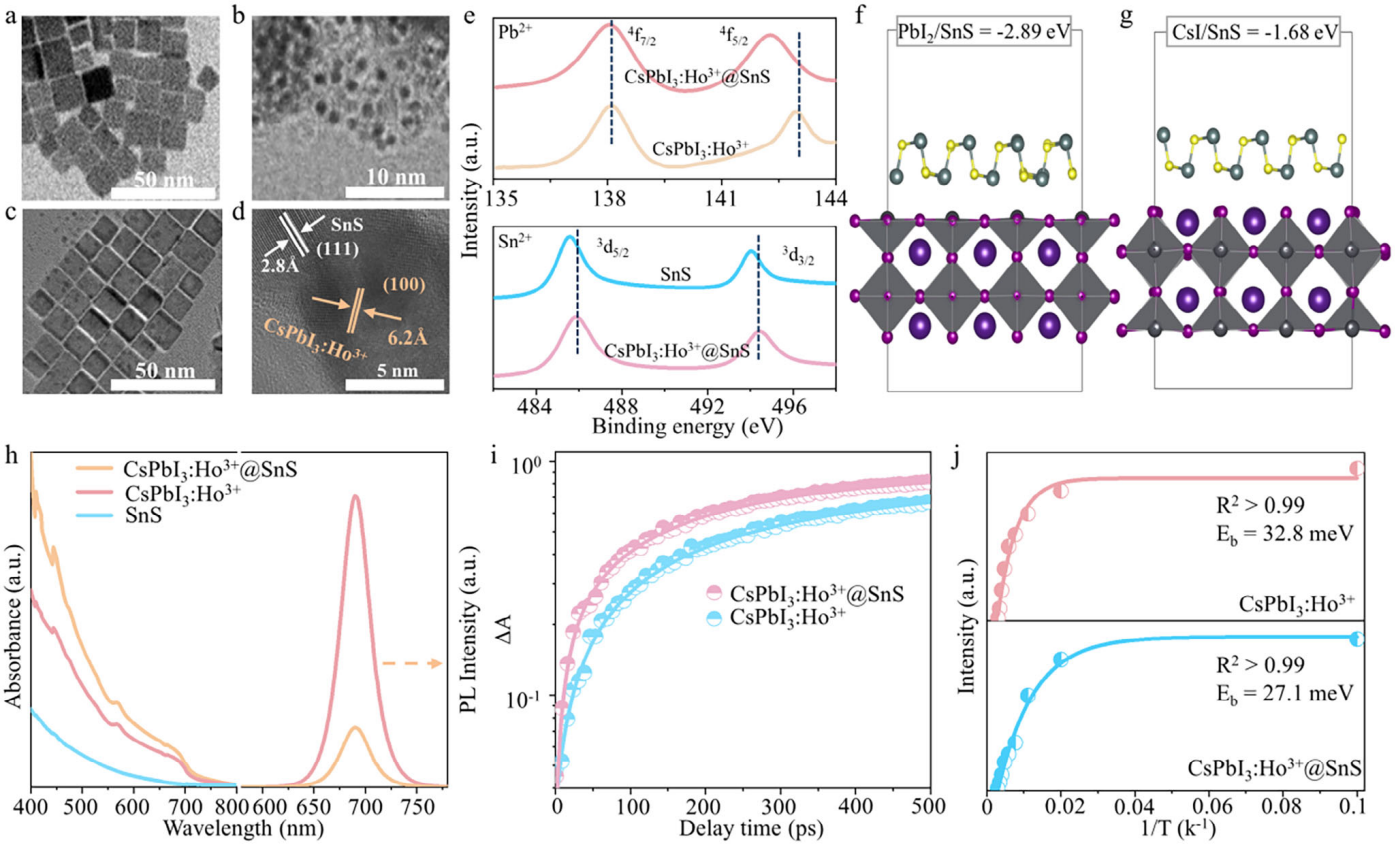
TEM images of CsPbI_3_:Ho^3+^ PQDs (a), SnS QDs (b), and CsPbI_3_:Ho^3+^@SnS QDs heterojunctions (c). d) HR‐TEM of CsPbI_3_:Ho^3+^@SnS QDs heterojunctions. e) XPS spectra of Pb 4*f* in CsPbI_3_:Ho^3+^ PQDs and CsPbI_3_:Ho^3+^@SnS QDs heterojunctions, and Sn 3*d* in SnS QDs and CsPbI_3_:Ho^3+^@SnS QDs heterojunctions. f, g) Optimized CsPbI_3_:Ho^3+^@SnS QDs heterojunctions with PbI_2_/SnS and CsI/SnS interfaces. h) Absorption and PL emission spectra of SnS QDs, CsPbI_3_:Ho^3+^ PQDs, and CsPbI_3_:Ho^3+^@SnS QDs heterojunctions. i) TA kinetic traces of CsPbI_3_:Ho^3+^ PQDs and CsPbI_3_:Ho^3+^@SnS QDs heterojunctions. j) Integrated PL intensity of CsPbI_3_:Ho^3+^ PQDs and CsPbI_3_:Ho^3+^@SnS QDs heterojunctions as a function of temperature, respectively.

The Ho^3+^ doped CsPbI_3_ PQDs with the high PLQYs (93.5%) and narrow bandgap, as the light absorption layer were prepared via the hot injection method.^[^ ^34^ ^]^ 2D SnS QDs with S‐based functional groups were synthesized following the chemical precipitation method (Figure S3, Supporting Information).^[^ ^24^, ^35^ ^]^ Then, SnS QDs blended with the CsPbI_3_:Ho^3+^ PQDs by electrostatic interactions (Figures S4 and S5, Supporting Information), owing to the opposite zeta potentials (ζ) of SnS QDs (−7.4 mV) and CsPbI_3_:Ho^3+^ PQDs (2.3 mV). The transmission electron microscope (TEM) and high‐resolution TEM images of CsPbI_3_:Ho^3+^ PQDs, SnS QDs, and CsPbI_3_:Ho^3+^@SnS QDs heterojunctions are shown in **Figures** **2**a–d and S6 (Supporting Information), where the uniform morphology and the clear lattice spacing of CsPbI_3_:Ho^3+^ PQDs (100) and SnS QDs (111) can be identified. The main diffraction peaks of both SnS QDs and CsPbI_3_:Ho^3+^ PQDs were appeared in CsPbI_3_:Ho^3+^@SnS QDs heterojunctions (Figure S7, Supporting Information), in which the (100) diffraction plane of CsPbI_3_:Ho^3+^@SnS QDs heterojunctions was redshift about ≈0.08°. The X‐ray photoelectron spectra (XPS) show that the binding energy of Pb^2+^ 4*f* and I^−^ 3*d* in CsPbI_3_:Ho^3+^ PQDs decreases after SnS QDs coupling (Figure 2e; Figure S8, Supporting Information). Meanwhile, the peak of Sn^2+^ 3*d* moves to the higher binding energy in CsPbI_3_:Ho^3+^@SnS QDs heterojunctions (Figure S9, Supporting Information). Compared to the CsPbI_3_:Ho^3+^ PQDs, the C─O bond in CsPbI_3_:Ho^3+^@SnS QDs heterojunctions with significantly increased (Figure S10, Supporting Information), indicating that the carboxylcontaining ligands of SnS QDs was coordinated in the CsPbI_3_:Ho^3+^ PQDs surface.^[^ ^36^ ^]^ The adhesive energies of PbI_2_/SnS and CsI/SnS were −2.89 and −1.68 eV by DFT calculation (Figure 2f,g), respectively, confirming that the main interactions of CsPbI_3_:Ho^3+^@SnS QDs heterojunctions in PbI_2_/SnS interface,^[^ ^37^ ^]^ which is consistent with the blue shift binding energy of Pb^2+^. Moreover, the density of states for CsPbI_3_:Ho^3+^@SnS heterojunctions further reveals the strong interface interaction between S 2*p* orbitals of SnS QDs and Pb 6*p* and I 5*p* orbitals of CsPbI_3_:Ho^3+^ PQDs (Figure S11, Supporting Information).^[^ ^22^ ^]^

The absorption intensity of CsPbI_3_:Ho^3+^@SnS QDs heterojunctions shows a slight increase within 400–800 nm, compared with the pristine CsPbI_3_:Ho^3+^ PQDs (Figure 2h). Whereas, the emission of CsPbI_3_:Ho^3+^@SnS QDs heterojunctions significantly quenching, accompanying reduced emission lifetime from pristine CsPbI_3_:Ho^3+^ QD 122.5–99.6 ns (Figure S12, Supporting Information). The kinetics of the transient absorption (TA) signals at 680 nm in Figure 2i reveal that the fast and slow times of CsPbI_3_:Ho^3+^ PQDs (324.5 and 1069 ps) decrease to 251 and 782.6 ps for CsPbI_3_:Ho^3+^@SnS QDs heterojunctions, indicating the effective charge extraction after building heterojunction.^[^ ^38^, ^39^, ^40^ ^]^ The CsPbI_3_:Ho^3+^@SnS QDs heterojunctions exhibit a weaker exciton binding energy (*E_b_*) of 27.1 meV relative to the pristine CsPbI_3_:Ho^3+^ PQDs (32.8 meV) in Figure 2j and Note S2 (Supporting Information), further confirming the boost charge separation after SnS QDs coupling.^[^ ^41^, ^42^ ^]^ Furthermore, the CsPbI_3_:Ho^3+^@SnS QDs heterojunctions have good air‐ and UV‐stability, maintaining over 85% and 90.2% of the original PL intensity after 60 days and 24 h UV light irradiation (Figures S13 and S14, Supporting Information), which is agreement with the negative binding energy of PbI_2_/SnS and CsI/SnS interface.^[^ ^24^ ^]^

**Figure 3:**
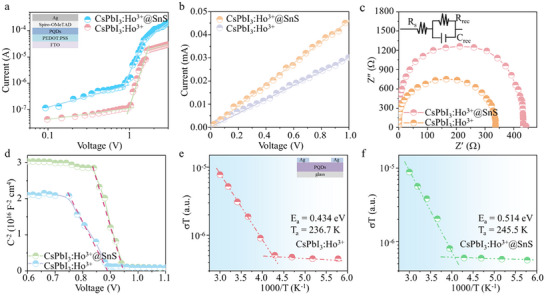
a) Current–voltage curves of the hole‐only devices based on CsPbI_3_:Ho^3+^ PQDs and CsPbI_3_:Ho^3+^@SnS QDs heterojunctions. b) *I*–*V* curves of CsPbI_3_:Ho^3+^ PQDs and CsPbI_3_:Ho^3+^@SnS QDs heterojunctions. Nyquist plots (c) and Mott–Schottky (d) of CsPbI_3_:Ho^3+^ PQDs and CsPbI_3_:Ho^3+^@SnS QDs heterojunctions. e, f) Temperature‐dependent conductivity of CsPbI_3_:Ho^3+^ PQDs and CsPbI_3_:Ho^3+^@SnS QDs heterojunctions.

The defect density can be obtained by the device of FTO/PEDOT:PSS/QDs/Spiro‐OMeTAD/Ag, as seen in **Figure** **3**a and Note S3 (Supporting Information). The values of CsPbI_3_:Ho^3+^ PQDs and CsPbI_3_:Ho^3+^@SnS QDs heterojunctions were calculated to be 5.08×10^16^ and 2.92×10^16^ cm^−3^, showing the effective trap passivation by SnS QDs modification. *I*–*V* curves and Nyquist plots in Figure 3b,c and Figure S15 (Supporting Information) demonstrate that the conductivity (σ) and the recombination resistance (*R_rec_*) of CsPbI_3_:Ho^3+^ PQDs were 3.54×10^−6^ S cm^−1^ and 335 Ω, while increase to 5.76×10^−6^ S cm^−1^ and 433 Ω for CsPbI_3_:Ho^3+^@SnS QDs heterojunctions, respectively. As shown in Figure 3d, the built‐in potential (V_bi_) of CsPbI_3_:Ho^3+^@SnS QDs heterojunctions (0.94 V) is larger than that of CsPbI_3_:Ho^3+^ PQDs (0.89 V). These results confirm that the defects in CsPbI_3_:Ho^3+^ PQDs are effectively passivated after SnS QDs capping, in line with the increased defect formation energy of I and Pb vacancies in heterojunctions. To further explore the performance of devices, the ions migration characteristics can be obtained by the Nernst–Einstein Equation^[^ ^32^ ^]^:

(1) $$\sigma \left(T\right)=\sigma_{0}/T\;\exp\;\left(-E_{a}/K_{b}T\right)$$

where *σ_0_* is constant, *E_a_* refers to the ion activation energy.

The *E_a_* of CsPbI_3_:Ho^3+^ PQDs and CsPbI_3_:Ho^3+^@SnS QDs heterojunctions were fitted to be 0.434 and 0.514 eV (Figure 3e,f), respectively. The larger *E_a_* indicates that vacancy and defect migration will be suppressed in devices. The conductivity transition temperature of CsPbI_3_:Ho^3+^ PQDs was 236.7 K, which increases to 245.5 K for CsPbI_3_:Ho^3+^@SnS QDs heterojunctions, benefiting the better stability of PDs.

**Figure 4:**
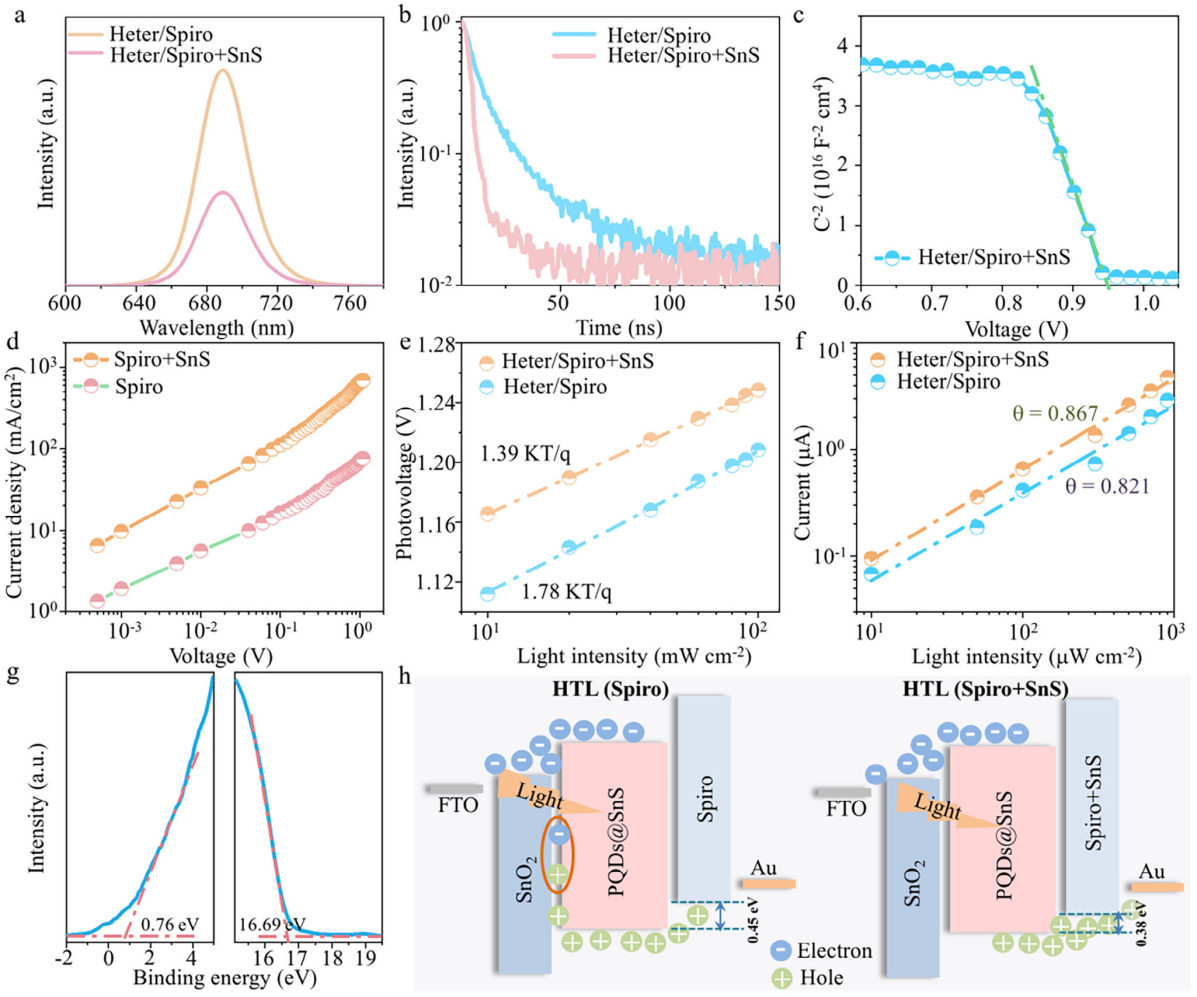
PL emission (a) and lifetime (b) curves of Heter/Spiro and Heter/Spiro+ SnS films. c) Mott–Schottky plot of Heter/Spiro+SnS film. d) *J*–*V* curves of hole‐only devices with FTO/PEDOT:PSS/Spior or Spiro+SnS/Au. Photovoltage (e) and current (f) dependence of light intensity curves for Heter/Spiro and Heter/Spiro+SnS. g) UPS plots of Spiro+SnS. h) Schematic of charge transfer for Heter/Spiro and Heter/Spiro+SnS devices.

Next, we further explore the hole extraction performance of Spiro with SnS doping. **Figure** **4**a displays the PL spectra of CsPbI_3_:Ho^3+^@SnS QDs heterojunctions/Spiro‐OMeTAD (Heter/Spiro) and CsPbI_3_:Ho^3+^@SnS QDs heterojunctions/SnS doped Spiro‐OMeTAD (Heter/Spiro+SnS). Compared with the Heter/Spiro, the emission intensity decreases for the Heter/Spiro+SnS film, indicating the more efficient hole extraction of Heter/Spiro+SnS HTL. Consistently, the PL lifetime decreases from 30.6 ns of the Heter/Spiro to 16.2 ns of Heter/Spiro+SnS (Figure 4b). The V_bi_ of Heter/Spiro+SnS (0.951 V) is larger than that of undoped devices (0.94 eV) in Figure 4c. Meanwhile, compared to the Spiro (6.25×10^−4^ cm^2^ V^−1^ S^−1^), the hole mobility of the Spiro+SnS increase to 8.31×10^−4^ cm^2^ V^−1^ S^−1^ in Figure 4d and Figure S16 (Supporting Information). Furthermore, the hole mobility of the Spiro+SnS is comparable to the electron mobility of SnO_2_ ETL (9.32×10^−4^ cm^2^ V^−1^ S^−1^), benefiting for the reduced interface hole accumulation and suppress electron‐hole recombination.

The photovoltage (V) depending on the light intensity (P) by fitting as follows:^[^ ^36^ ^]^

(2) $$V\left(P\right)=\frac{nk_{b}T}{q}\ln\;\left(P\right)+C$$

where n and C are the ideal factor and the constant.

It is found that the Heter/Spiro+SnS with larger V than that of the Heter/Spiro (Figure 4e). Moreover, compared to the Heter/Spiro (1.78 *kT*/*q*), the slope of the Heter/Spiro+SnS device decreases to 1.39 *kT*/*q*. The *I*–*P* curves of the Heter/Spiro and Heter/Spiro+SnS are shown in Figure 4f. By the law:^[^ ^43^ ^]^ *I_p_∝P^α^*, where α of Heter/Spiro+SnS (0.867) is larger than that of Heter/Spiro (0.821), leading to the high detection of the self‐powered PDs. The Fermi level and VBM of Spiro+SnS (−4.53 and −5.29 eV) are lower than that of pristine Spiro (−4.42 and −5.22 eV) in Figure 4g, providing an appropriate energy level to improve the hole transport for Heter/HTL interface. Figure 4h schematically presents the charge transfer mechanism of the PDs with and without SnS QDs doping. For the PDs with the pristine Spiro HTL, a part of hole accumulation at the Heter/ETL interface because of the unbalanced electron and hole mobilities, and imperfect energy band alignments, resulting in larger Auger recombination.^[^ ^44^ ^]^ After SnS QDs doped into Spiro HTL, the relatively balanced ETL and HTL mobility and the more appropriate energy level of the Heter/HTL interface, led to efficient hole extraction and transmission and improved the performance of self‐powered PDs.

**Figure 5:**
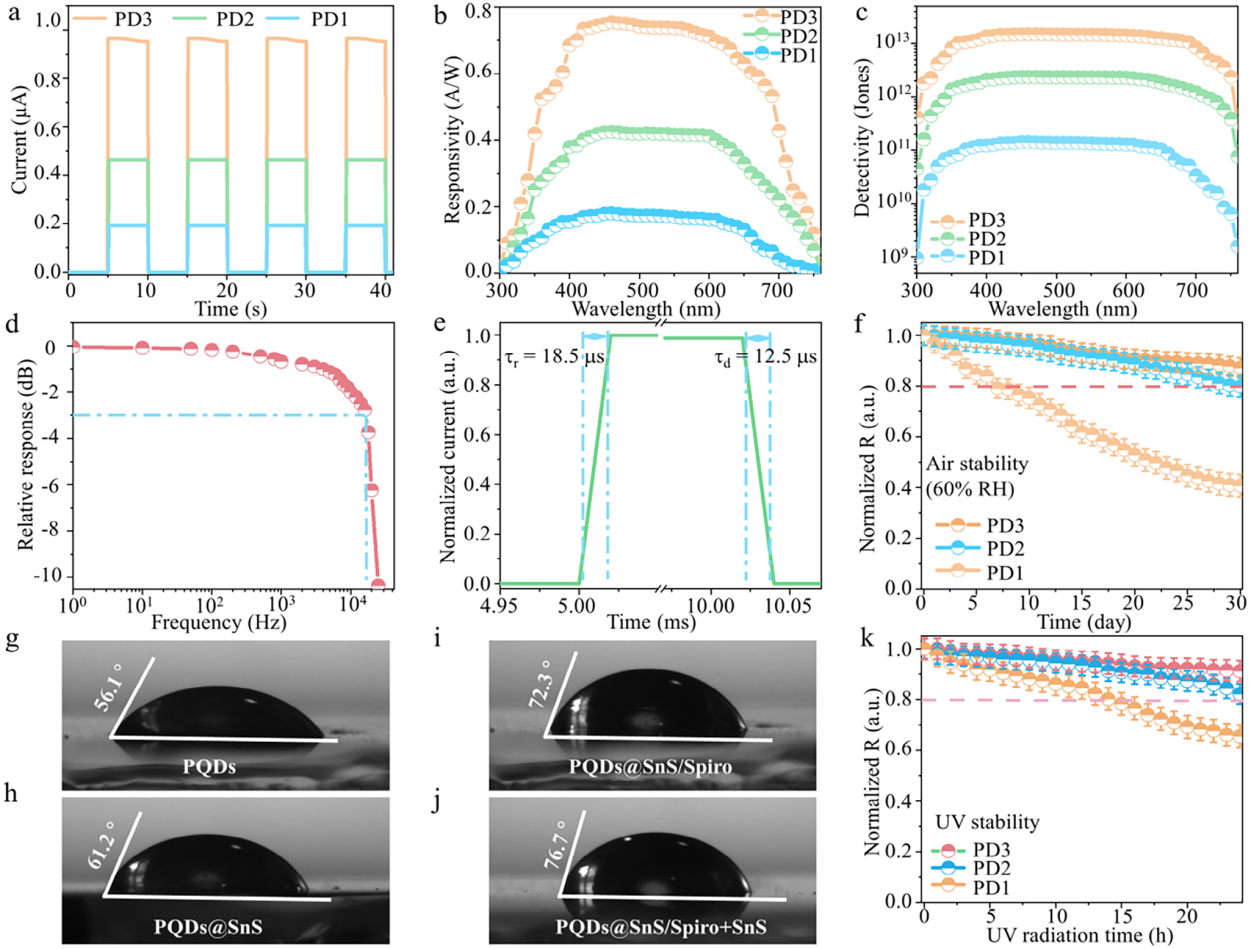
a) *I*_p_–*t* curves of the PD1‐PD3 under 460 nm light illumination with 0 V bias. b, c) Responsivity and detectivity of the PD1‐PD3. d) Relative response with the modulation frequency measured for PD3. e) Rise and decay times for PD3. f) Normalized R of the PD1‐ PD3 after 30 days under 60% RH. Water contact angles of CsPbI_3_:Ho^3+^ PQDs (g), CsPbI_3_:Ho^3+^@SnS QDs heterojunctions (h), CsPbI_3_:Ho^3+^@SnS QDs heterojunctions/Spiro (i), and CsPbI_3_:Ho^3+^@SnS QDs heterojunctions/Spiro+SnS (j). k) Normalized R of the PD1‐PD3 after 24 h UV light irradiation.

The photocurrent–time (*I*_p_–*t*) curves of the FTO/SnO_2_/CsPbI_3_:Ho^3+^ PQDs/Spiro/Au (PD1), FTO/SnO_2_/CsPbI_3_:Ho^3+^@SnS QDs heterojunctions/Spiro/Au (PD2), and FTO/SnO_2_/CsPbI_3_:Ho^3+^@SnS QDs heterojunctions/Spiro+SnS/Au (PD3) under the 460 nm light illuminate with 0 V bias were measured in **Figure** **5**a. The I_p_ of PD1 was ≈0.182 µA, largely improving to 0.491 and 0.974 µA for PD2 and PD3, respectively. The dark currents (I_d_) of the PD3 (1.08 pA) were lower than that of the control PD1 (0.601 nA), which indicates that the defects of CsPbI_3_:Ho^3+^ PQDs were passivized and more favorable charge separation by SnS QDs coupling, and efficient hole extraction and transportation for Spiro+SnS HTL.^[^ ^11^, ^45^ ^]^ The responsivity (R), external quantum efficiency (EQE), and detectivity (D*) of PD1‐PD3 were obtained in Figure 5b,c, Figure S17 and Note S4 (Supporting Information). The R, EQE, and D* of PD3 were 0.76 A W^−1^, 90.6% and 1.51×10^13^ Jones at the 460 nm, respectively. Compared to PD1, the R, EQE, and D* of PD3 increase by 4.19 folds, 1.87 folds, and 106 folds. The R and D* distribution from 100 devices are shown in Figures S18 and S19 (Supporting Information), demonstrating the highest performance for PD3. As seen in Figure 5d, the −3 dB bandwidth of PD3 was 1.61×10^4^ Hz under 460 nm light, indicating a rapid light response. According to the law:^[^ ^46^ ^]^ f_‐3dB_ = 0.35/τ, the response time (τ) of PD3 is estimated to be 21.7 µs. In addition, τ of the PD1‐PD3 were also tested by the standard square wave method (Figure 5e; Figure S20, Supporting Information), where the PD3 displays the fastest rise time of 18.5 µs and decay time of 12.5 µs. The linear dynamic range (LDR) of the device is 77.3 (Figure S21, Supporting Information), which is much larger than that of other reported.

To further evaluate the stability of self‐powered broadband PDs with SnS QDs incorporating, the R of PD1‐PD3 as a function of humidity stability was tested. Figure 5f shows the R of PD1‐PD3 under ambient air (RH = 60%) after 30 days storage, in which PD3 retains ≈87.2% of its original R, while the PD1 falls quickly and decreases to less than 40% of the initial R. The water droplet contact angle of CsPbI_3_:Ho^3+^ PQDs, CsPbI_3_:Ho^3+^@SnS QDs heterojunctions, CsPbI_3_:Ho^3+^@SnS QDs heterojunctions/Spiro, and CsPbI_3_:Ho^3+^@SnS QDs heterojunctions/Spiro+SnS were 56.1°, 61.2°, 72.3°, and 76.7° in Figure 5g–j, respectively, suggesting the improved hydrophobicity after cooperating SnS QDs with CsPbI_3_:Ho^3+^ PQDs and Spiro HTL. Meanwhile, the R of the PD3 still remains ≈91.2% of the original value after 24 h UV light illumination, whereas the PD1 only kept 65.5% for its initial R (Figure 5k). Furthermore, the R of PDs remained at 94.5% for the original R‐value after 180 h storage under humidity conditions (Figure S22, Supporting Information).

**Figure 6:**
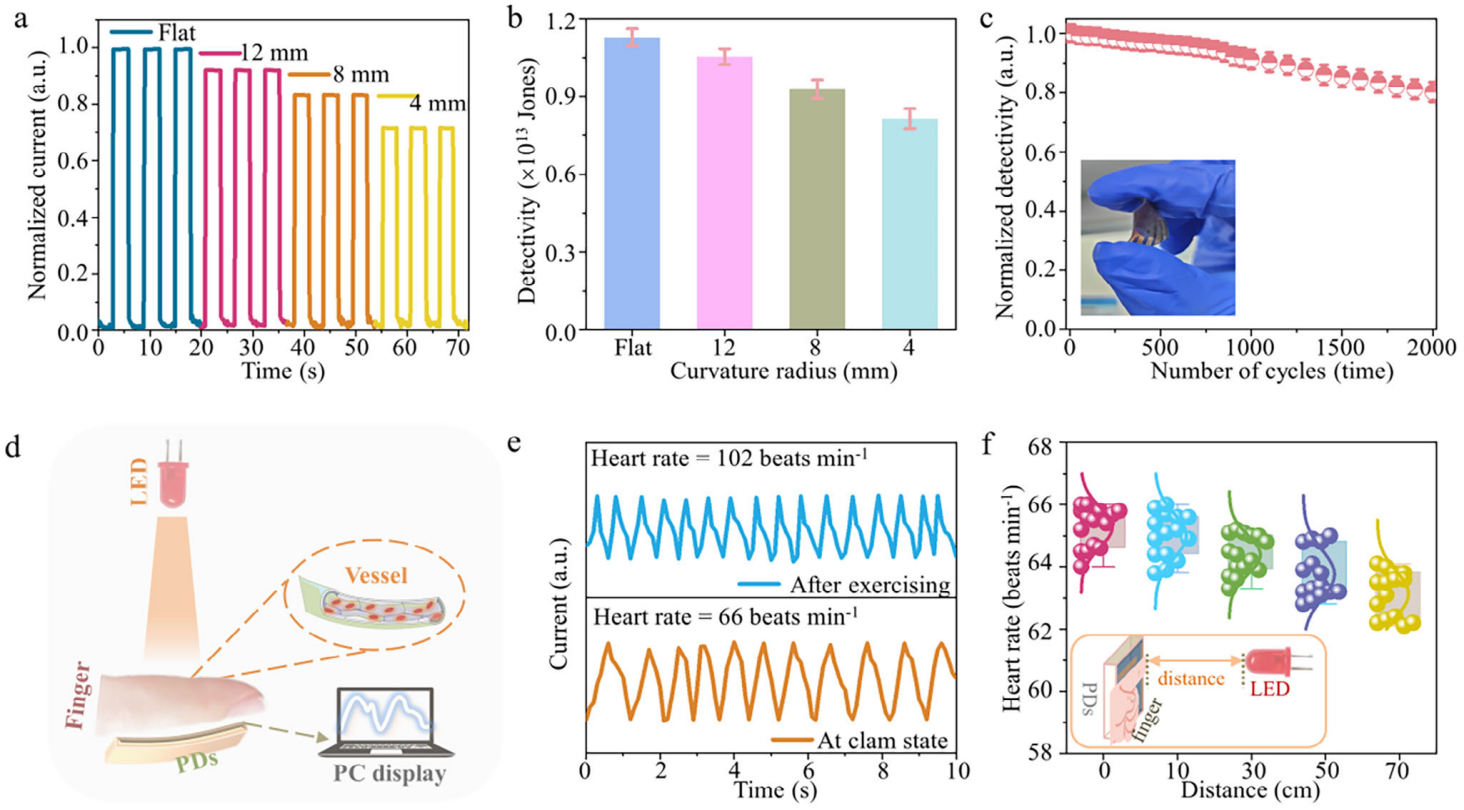
a) Normalized *I*_p_–*t* curves of the flexible self‐powered PDs after flexing at a curvature radius of 0, 12, 8, and 4 mm. b) Detectivity of the flexible self‐powered PDs after flexing at a curvature radius of 0, 12, 8, and 4 mm. c) Normalized detectivity of flexible self‐powered PDs at a curvature radius of 4 mm after 2000 cycles. The inset shows the device photos of the flexible PDs film. d) Schematic diagram of heart rate monitoring by the flexible self‐powered PDs and 710 nm LED illumination. e) Current response at clam state and after exercise by this device. f) Heart rate signal measured with the different distances between LEDs and fingers.

Then, a flexible self‐powered broadband PDs based on the PET substrate was fabricated, and the *I*_p_–*t* curves of these PDs with the different curvature radii under 460 nm light illuminate are shown in **Figure** **6**a. It can be seen that the I_p_ still maintains 83.2% and 72.6% of the initial current value after the curvature radius decreases to 8 and 4 mm, demonstrating good flexible response performances and excellent repeatability. The D*, R, and τ of the flexible self‐powered PDs were obtained to be 1.13×10^13^ Jones, 0.58 A W^−1^, and 98.8 µs, respectively (Figure 6b; Figures S23–S25, Supporting Information). Importantly, the D* and R of the device can obtain a higher value of 0.815×10^13^ Jones and 0.41 A W^−1^ even though the curvature radius decreases to 4 mm. Moreover, the D* of the flexible self‐powered PDs still remains at 80.2% of the initial value under the radius of 4 mm after 2000 bending cycles (Figure 6c), which exceeds the most results of the previous reports. **Table** **1** shows the key parameters of flexible self‐powered PDs, suggesting that our PDs have better performances with the higher R, D*, and τ values.

**Table 1:** Performance summary of the flexible self‐powered PDs.

Materials	Wavelength (nm)		R[A W^−1^]	D*[Jones]	τ_r_/τ_d_ [µs]	Refs.
D18‐Cl/Y6		400–950	0.47	3.63×10^12^	81	[47]
Si/CdS		320–1550	0.34×10^−3^	1.3×10^12^	492	[48]
CQDs/MAPbI_3_		350–800	0.42	8.4×10^12^	0.96	[49]
CsPbI_3‐x_Br_x_		350–650	‐	8.9×10^9^	‐	[50]
Ag/rGO/Cu		400–1600	5.5×10^−3^	1×10^9^	‐	[51]
*n*‐MoS_2_ QD/*n*‐MoS_2_/*p*‐CuO		376–1064	0.83×10^−4^	1.46×10^10^	7.736×10^4^	[52]
organic rubrene crystal/graphene		300–820	8×10^5^	>10^12^	21/120	[53]
MAPbI_3_		350–800	0.2	2×10^11^	3.9×10^4^	[54]
γ‐InSe		300–850	2.49×10^−2^	4.9×10^10^	222/288	[55]
CuI/a‐ZTO		405–635	‐	3.77×10^11^	800/600	[56]
FA_0.92_Cs_0.04_MA_0.04_PbI_3_		350–800	0.55	8.3×10^9^	3.5×10^4^	[57]
MAPbI_3_/PCBM		300–850	0.12	‐	9×10^3^	[10]
SnO_2_/CsPbBr_3_/carbon		350–550	0.15	3.7×10^11^	110/230	[58]
D18:Y6		300–1000	0.35	1.14×10^12^	‐	[9]
MXene/PbS		470–980	1.15×10^−2^	2.4×10^11^	3×10^4^	[59]
CsPbI_3_:Ho^3+^@SnS/Spiro+SnS		300–750	0.58	1.13×10^13^	98.8	this work

Based on the outstanding performance of the flexible self‐powered PDs, we further integrate them with the LEDs for real‐time heart rate monitoring. As seen in Figure 6d, the finger was placed between flexible self‐powered PDs and 710 nm LEDs, and the flexible self‐powered PDs can convert the pulse light signal to the current. It found that the heart rate of ≈66 beats min^−1^ at calm state, and ≈102 beats min^−1^ after exercise (Figure 6e). The heart rate changes after exercise by the flexible self‐powered PDs and commercial PPG device are shown in Figure S26 (Supporting Information), where the data is almost identical to our device and commercial PPG system. Meanwhile, alcohol intake can lead to heart rate variations, particularly for individuals with poor health conditions.^[^ ^6^ ^]^ Figure S27 (Supporting Information) shows the heart rate change with the increased alcohol intake levels, where it can be calculated to ≈72 beats min^−1^ with 41.6 g alcohol, and ≈90 beats min^−1^ with 83.2 g alcohol, exhibiting better measurement precision by this device. Besides, the flexible self‐powered PDs are promising for remote heart rate monitoring, owing to the high signal‐to‐noise ratio (≈9.02×10^5^). As the monitoring distance increased to 10, 30, 50, and 70 cm, the heart rate was ≈65, ≈64, ≈64, and ≈63 beats min^−1^ (Figure 6f), respectively, showing the accurate remote monitoring. In addition, this device demonstrates exceptional practical application, where the heart rate monitoring accuracy almost no changes after 60 days of storage (Figure S28, Supporting Information).

## 3 Conclusion

In summary, the high performance and stability flexible self‐powered PDs can be successfully fabricated based on the CsPbI_3_:Ho^3+^@SnS QDs type II *p*‐*n* heterojunction and a composite HTL by SnS QDs doped into spiro‐OMeTAD, showing the responsivity of 0.58 A W^−1^, detectivity of 1.13×10^13^ Jones, and response time of 98.8 µs under 0 bias, respectively. The optimized CsPbI_3_:Ho^3+^@SnS QDs type II *p*‐*n* heterojunction demonstrated low trap density, large built‐in field, and effective charge separation compared to the pristine PQDs. Meanwhile, based on the suitable energy band level and high hole mobility of SnS QDs, the SnS QDs doped into a spiro‐OMeTAD layer, leading to improved hole extraction and transportation, balanced electron and hole mobilities, and restrained interface recombination. Finally, a wearable PPG system was realized by these flexible self‐powered PDs and LEDs, enabling real‐time and accurate heart rate monitoring.

## Conflict of Interest

The authors declare no conflict of interest.

## Supporting information



Supporting Information

## Data Availability

The data that support the findings of this study are available from the corresponding author upon reasonable request.
