# Supplementary material for: Heterojunction Derived Efficient Charge Separation for High Sensitivity Self‐Powered Flexible Photodetectors toward Real‐Time Heart Rate Monitoring
Source: Adv Sci (Weinh). 2025 May 24;12(28):2505945. doi: 10.1002/advs.202505945 (PMC12302588; doi:10.1002/advs.202505945)
Supplement: Supplementary file 1 — Supporting Information [file ADVS-12-2505945-s001.docx]

**Heterojunction Derived Efficient Charge Separation for High Sensitivity Self-powered Flexible Photodetectors Toward Real-time Heart Rate Monitoring**

*Nan Ding,^1^ Ge Zhu,^1^ Xiaotao Zhang,^2,3^ Wen Xu,^1^* Hailong Liu,^1^ Yanan Ji,^1^ Yuanzheng Chen,^2^* Bin Dong^1^**

1. *Key Laboratory of New Energy and Rare Earth Resource Utilization of State Ethnic Affairs Commission, School of Physics and Materials Engineering, Dalian Minzu University, Dalian 116600, China.*
2. *School of Physical Science and Technology, Southwest Jiaotong University, Chengdu 610031, China.*
3. *Zhuhai Beijing Institute of Technology (BIT), Beijing Institute of Technology, Zhuhai 519088, China*

*^*^E-mail: Prof. Wen Xu* [*(xuwen@dlnu.edu.cn),*](mailto:(xuwen@dlnu.edu.cn),) *Prof. Yuanzheng Chen (*[*cyz@swjtu.edu.cn*](mailto:cyz@swjtu.edu.cn)*), Prof. Bin Dong (dong@dlnu.edu.cn).*

**Materials:**

Cs_2_CO_3_ (99.9%), octadecene (ODE, 90%), oleic acid (OA, 90%), oleylamine (OAm, 70%), PbI_2_ (99.999%), HoI_3_ 6H_2_O (99.99%), SnCl_2_ (99.9%), thioacetamide (Aldrich), TOP (technical grade 90%, Aldrich), and toluene (ACS grade, Fischer) were all purchased from Sigma-Aldrich and used as starting materials without further purification. Spiro-OMeTAD (99.5%) was purchased from Youxuankeji. 4-tert-butylpyridine (t-BuPy), acetonitrile (ACN) (>99.9%), and lithiumbis (triﬂuoromethanesulfonyl) imide (Li-TFSI) were obtained from Aladdin.

**Synthesis of CsPbI_3_:Ho^3+^ PQDs***:*

Firstly, 0.8 g of Cs_2_CO_3_ was loaded into a mixture of 30 mL of ODE and 2.5 mL of OA and then heated to 120 °C until completely dissolved. Then, the mixture was kept at 120 °C for 1 h. Note that, during the synthesis of perovskite QDs, the temperature of Cs-oleate mixture should be kept at least at 130 °C to avoid precipitation. PbI_2_ (0.5 mmol), HoI_3_ (0.3 mmol), OAm (1.5 mL), OA (1 mL), and ODE (10 mL) were added to a 3-neck round bottomed flask and were evacuated and refilled with N_2_ followed by heating the solution to 120 °C for 1 hour. Then, the solution was then increased to 200 °C for 10 minutes each. Finally, the Cs-oleate (1 mL) was swiftly injected and after 10 s the solution was cooled with an ice bath. The PQDs were precipitated with acetone and the centrifuged followed by dissolving in hexanes.

**Synthesis of SnS QDs:**

5 mL of ODE, 3 mL of TOP, 4.5 mL of OA, and 0.383 g of SnCl_2_ were loaded into a 100 mL three-necked flask under 120 °C for 1 h with nitrogen condition. Then, the mixture was heated to 150°C and kept 30 min. The 10 mL of OAm, 3 mL of TOP, and 0.075 g of thioacetamide were quickly injected. The reaction was quenched after 5 min. The purification QDs were transferred to the glovebox for further use.

**Preparation of the CsPbI_3_:Ho^3+^@SnS QDs p-n heterojunctions composite:**

In a typical procedure, the CsPbI_3_:Ho^3+^ solution (10 mL) was mixed with 2 mL of SnS QDs toluene solution. Under dark conditions, the suspension was ultrasonicated for 10 min and stirred for 3 h. Finally, the CsPbI_3_:Ho^3+^@SnS heterojunction composites were collected by centrifuging at 8000 rpm for 5 min.

**Device fabrication:**

The FTO substrates were cleaned with deionized water, ethanol, acetone and isopropanol, and were finally treated under oxygen plasma for 20 min to remove organic residues. SnO_2_ layers were deposited by spin coating a solution at a speed of 5000 rpm and annealing in air at 150 °C for 30s. Then, the FTO substrates were transferred into glovebox. Then, 15 µL of as-prepared solution of CsPbI_3_:Ho^3+^@SnS QDs p-n heterojunctions was spin coated on the FTO/ SnO_2_ substrate at 1000 rpm for 15 s and 2000 rpm for 20 s. 150 uL of MeOAc was dropped on the CsPbI_3_:Ho^3+^@SnS QDs p-n heterojunctions layer for 5 s to remove the long chain insulated ligands at the surface of PQD and then spun at 2000 rpm for 20 s. This process was needed to repeat five times to obtain thick enough CsPbI_3_:Ho^3+^@SnS QDs p-n heterojunctions film. For the hole-transport layer, it was obtained by spin coating the SnS QDs doped Spiro-OMeTAD solution at 3000 rpm for 30 s. At last, on top of the Spiro-OMeTAD layer, the Au electrode was thermally evaporated. For ﬂexible PDs, which can be fabricated on ﬂexible PET substrate used the same methods.

**Characterization:**

The photocurrent-time curves were tested by a Keithley 2400 source-meter. The HR-TEM images were characterized by using a JEOL-TEM high-resolution transmission electron microscope. SEM was carried out by a SIRION field-emission scanning electron microscope at 5 kV. The UV-vis absorption spectra were recorded by a UV-3600 (Shimadzu, Japan) spectrophotometer. Photoluminescence and excitation spectra were measured by RF-6000 (Shimadzu, Japan). UPS data were obtained by ThermoFisher ESCALAB 250Xi from Shiyanjia Lab. Powder XRD was obtained on Rigaku D/max 2550 X-ray diffractometer with Kα radiation of Cu at a scanning speed of 15° min-1 and operated at 8 kW. AFM topography was characterized using an Agilent 5500 scanning probe microscopy operated in contact mode. EIS measurement was recorded with AMETEK Versa STAT 4 at a frequency of 0.1 Hz-0.1 MHz in the dark. The plane-wave energy cutoff was set to 550 eV, and the Monkhorst-Pack method was employed for the Brillouin zone sampling. The convergence criteria of energy and force calculations were set to 10-5 eV/atom and 0.03 eV/Å, respectively. Time-resolved photoluminescence (TRPL) spectrum was measured using an Edinburgh Instruments FS980 system. The PLQYs of films were obtained by fluorescence spectrometer (Edinburgh). The XPS spectra were measured using a an EDAX Inc. For the device analysis, Xenon lamp was used to illuminate the device. The electrical property of the PDs or thin film were carried out on a semiconductor measuring setup (4200-SCS, Keithley Co. Ltd.).

**Supplementary Note 1**

**Computational methods:**

All DFT calculations were performed using the projector augmented wave (PAW) method with the consideration of spin-polarization, as implemented in the Vienna ab initio Simulation Package (VASP). The exchange-correction functional was approximated by the Perdew-Burke-Ernzerhof (PBE) functional within the generalizes gradient approximation (GGA) method. In structural optimizations, the cutoff energy of the plane wave basis was set to 520 eV. The convergence standards of energies and forces were set to 10^-5^ eV and 0.002 eV/Å, respectively. Besides, a 4 × 4 × 4 and 6 × 6 × 2 gamma-centered k-points mesh grid in Brillouin zone were adopted for the unit cell of CsPbI_3_ and SnS. The heterojunctions lattice constant is a = 19.4 Å and b = 18.9 Å. The vacuum layer is set to be 30 Å to avoid the interactions between periodic images.

The adhesive energy can be determined using the following equation:

$$E_{\mathrm{ad}}=E_{\mathrm{hete}}- E_{\mathrm{SnS}}-E_{\mathrm{CsPb}I_{3}}$$

Where the $E_{\mathrm{hete}}$, $E_{\mathrm{SnS}}$, and $E_{\mathrm{CsPb}I_{3}}$ represents the total energy of the heterojunctions, the corresponding monolayer SnS and CsPbI_3_ slab, respectively. CsPbI_3_ slab with different interfaces have different values in $E_{\mathrm{CsPb}I_{3}}$.

The charge density difference can be obtained by the following:

$$\Delta\rho= \rho_{\mathrm{AB}}- \rho_{A}-\rho_{B}$$

Where $\rho_{\mathrm{AB}}$, $\rho_{A}$ and $\rho_{B}$ are the charge densities of the heterojunctions, the slab A and the slab B, respectively.

The defect formation energy $E_{f}$ is expressed as follows:

$$E_{f}=E_{D}- E_{p}+N\mu_{I}+M\mu_{\mathrm{Pb}}$$

Where $E_{D}$ and $E_{p}$ are the energies of the structure with and without the vacancies. N and M are the numbers of the defective I and Pb atoms in the structure, while $\mu_{I}$ and $\mu_{\mathrm{Pb}}$ represent the chemical potential of single I and Pb atoms. Besides, $\mu_{I}$ and $\mu_{\mathrm{Pb}}$ are constrained by the relationship in the CsPbI_3_ crystal:

$$\Delta H=E\left( \mathrm{CsPb}I_{3} \right)-\left( E\left( \mathrm{Cs} \right) + E\left( \mathrm{Pb} \right) +3E\left( I \right) \right)= \mu_{\mathrm{Cs}}+\mu_{\mathrm{Pb}}+ 3\mu_{I}$$

Where $\Delta H$ is formation entropy of $\mathrm{CsPb}I_{3}$. And $E\left( \mathrm{CsPb}I_{3} \right)$, $E\left( \mathrm{Pb} \right)$ and $E\left( I \right)$ are the total energies of per unit of $\mathrm{CsPb}I_{3}$ cell, Pb bulk and I bulk respectively. And the $E\left( \mathrm{Cs} \right)$ is assumed to be equal to $\mu_{\mathrm{Cs}}$. For I-rich conditions, the $\mu_{I}$ can be regarded as 0. For Pb-rich conditions, the $\mu_{Pb}=0$.

**Supplementary Note 2**

The temperature dependent PL intensity of CsPbI_3_:Ho^3+^ PQDs and CsPbI_3_:Ho^3+^@SnS QDs type-II p-n heterojunctions were calculated as follows:

$I_{T}$= $\frac{I_{0}}{1+Ae^{-E_{b}{/K}_{b}T}}$

where *I_0_* and *I_T_* presents the PL intensity of PQDs at 0 K and temperature *T*, *A* and *K_b_* refers to the proportional constant and Boltzmann constant, *E_b_* is the exciton binding energy. The full width at half-maximum (FWHM) as a function of temperature can be fitted by:

*Γ(T) = Γ_0_ + σT +* $\frac{{}_{OP}}{e^{(E_{op}/KT)}-1}$

where *𝛤_0_* is the temperature independent term induced by disorder and imperfections at 0 K. *Γ(T), E_op_,* and *Γop* are the FHWM of PQDs at temperature *T,* the energy of optical phonons, and the coupling coefficient of the interactions between excitons and optical phonons.

**Supplementary Note 3**

The defect density (N_t_) and the carrier mobility (μ) of CsPbI_3_:Ho^3+^ PQDs and CsPbI_3_:Ho^3+^ PQDs@SnS QDs are obtained in FTO / PEDOT:PSS / PQDs / Spiro-OMeTAD / Ag devices. They can be estimated as follows:

$N_{t}$ = $\frac{2_{0}V_{TFL}}{{eL}^{2}}$

μ = $\frac{{8JL}^{3}}{9{}_{0}V^{2}}$

where V*_TFL_* is the trap-filled limit voltage,$L$ is the thickness of the perovskite film, ε_0_ and ε are the vacuum permittivity and the relative dielectric constant of PQDs bulk material, J and V are the current density and the applied voltage.

**Supplementary Note 4**

These parameters satisfy the following equations:

R=$\frac{I_{ph}-I_{d}}{PS}$

D* =$\sqrt{\frac{S}{{2eI}_{d}}}$R

EQE= R$\frac{hc}{e}$

where $I_{ph}$and $I_{d}$ are the photocurrent under the illumination of light and in the dark, $P$ and $S$ are the input light power density and the effective irradiated area (0.1×0.1 cm^2^), $h$ and $c$ are the Planck’s constant and the speed of light, and $e$ are the incident light wavelength and the elementary charge.


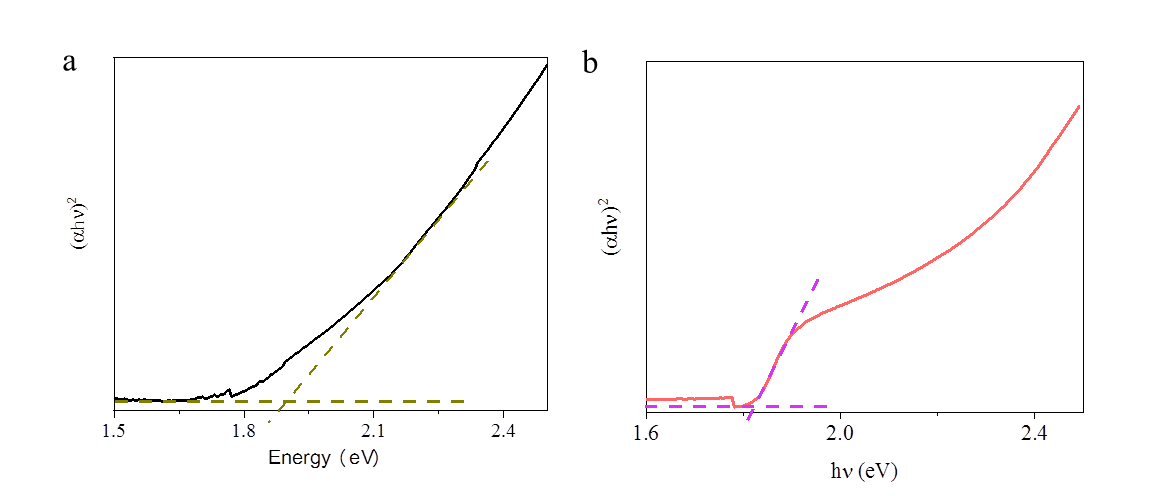


Figure S1. The bandgaps of SnS QDs (a) and CsPbI_3_:Ho^3+^ PQDs (b).


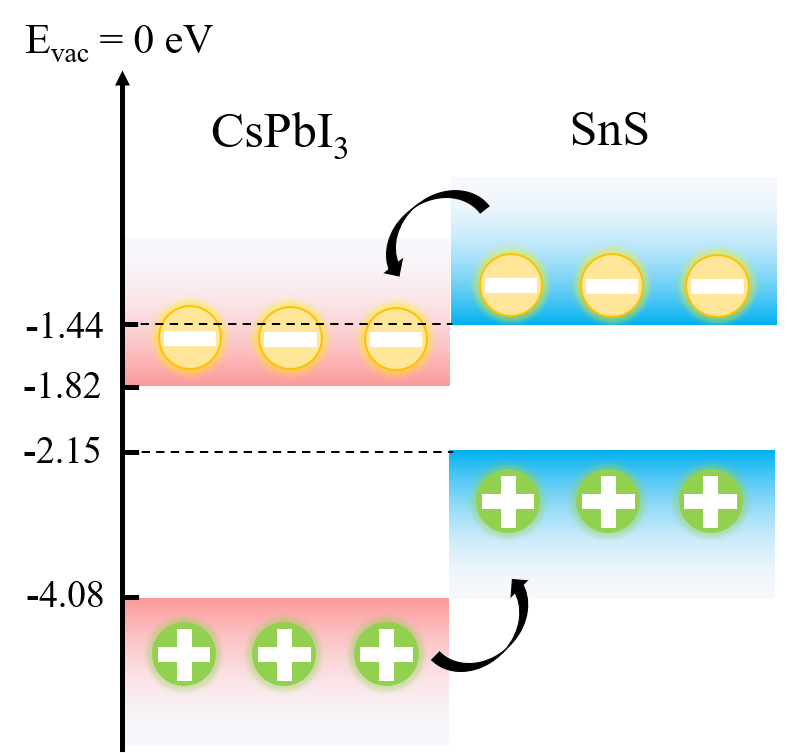


Figure S2. Schematic of the type-II band alignment of the CsPbI_3_:Ho^3+^@SnS heterojunction. The vacuum level of CsPbI_3_:Ho^3+^ and SnS (E_vac_) are aligned and set to be 0 eV.


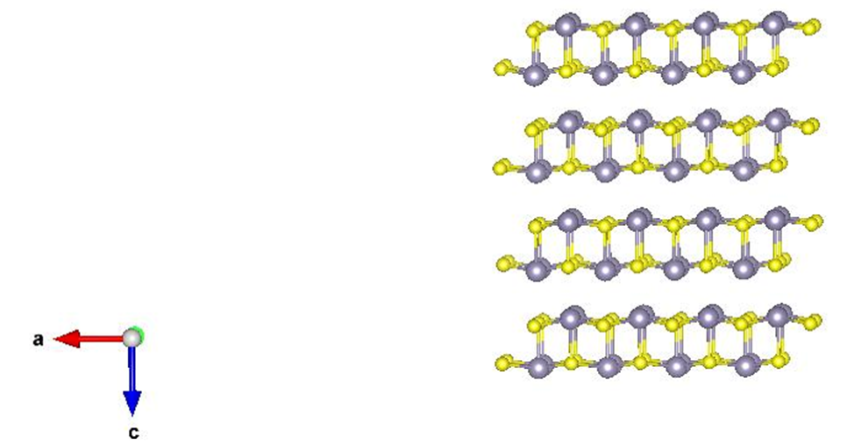


Figure S3. Structural diagram of SnS QDs.


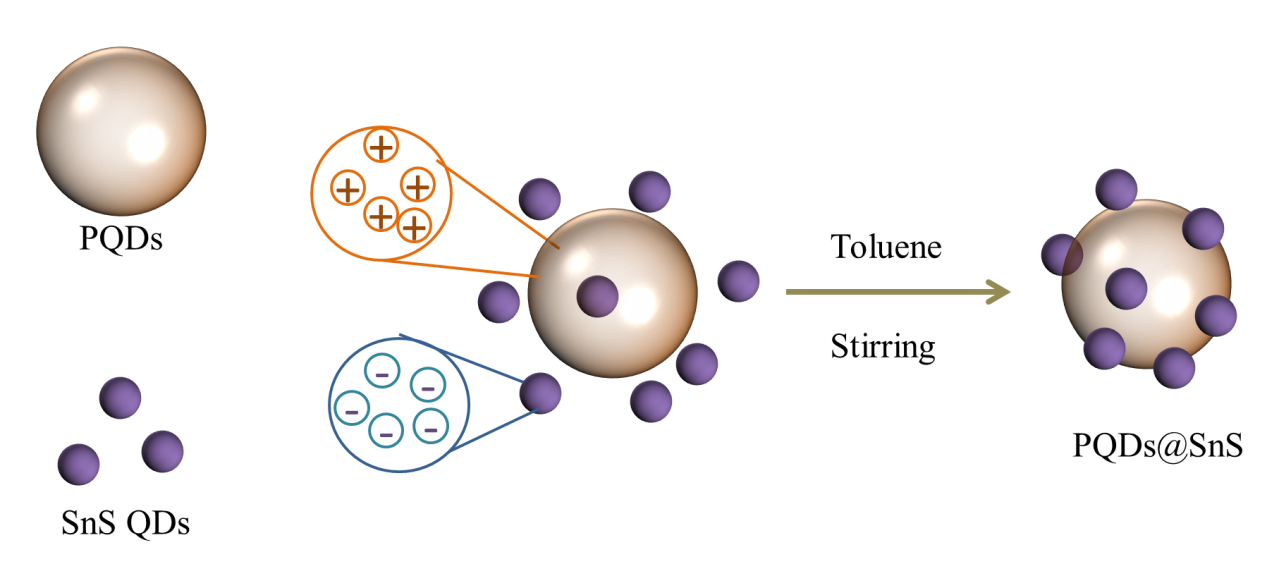


Figure S4. Schematic illustration of the assembly of the CsPbI_3_:Ho^3+^@SnS heterostructures QDs.


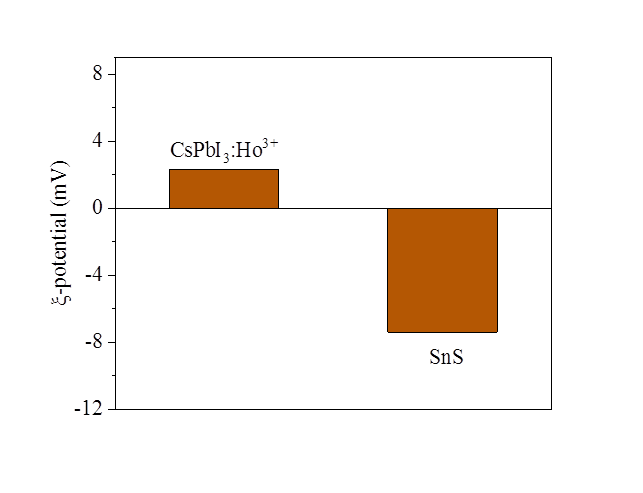


Figure S5. Variations in the zeta (ζ) potentials of CsPbI_3_:Ho^3+^ PQDs and SnS QDs.


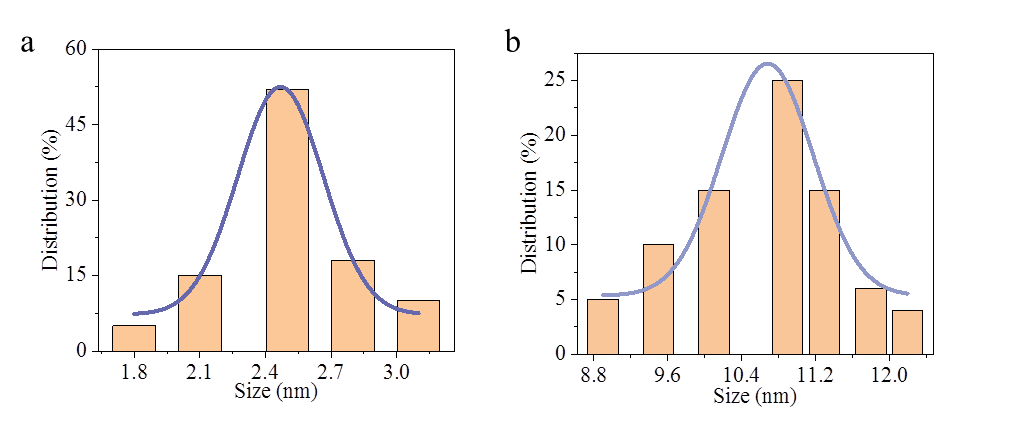


Figure S6. The average diameter of SnS QDs.


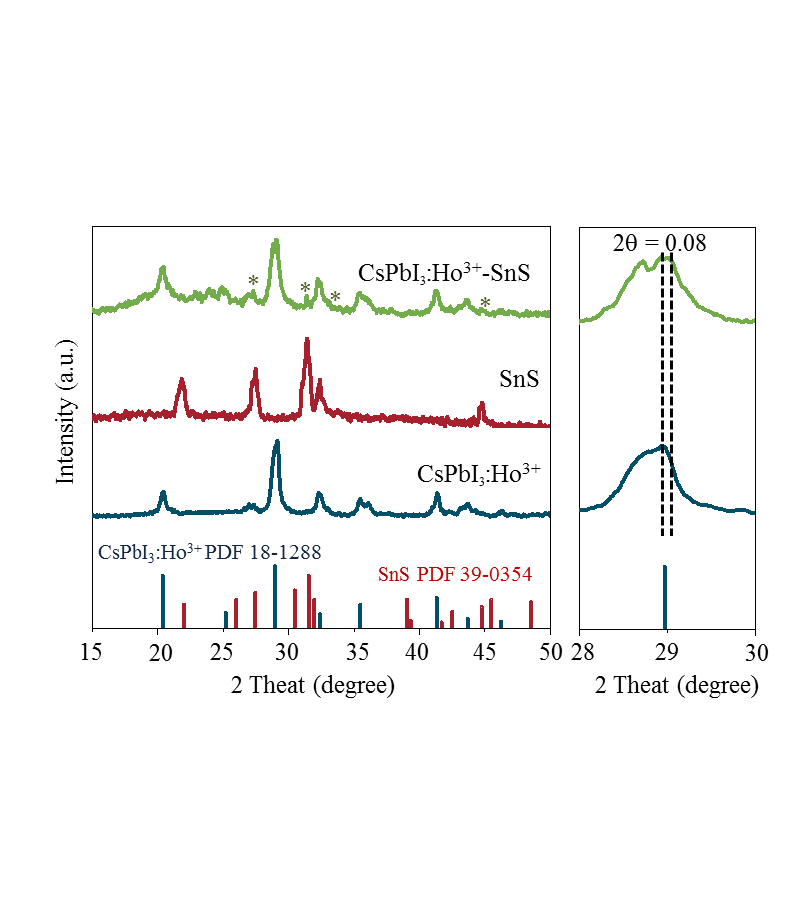


Figure S7. XRD patterns of CsPbI_3_:Ho^3+^ PQDs, SnS QDs, and CsPbI_3_:Ho^3+^@SnS heterostructures QDs.


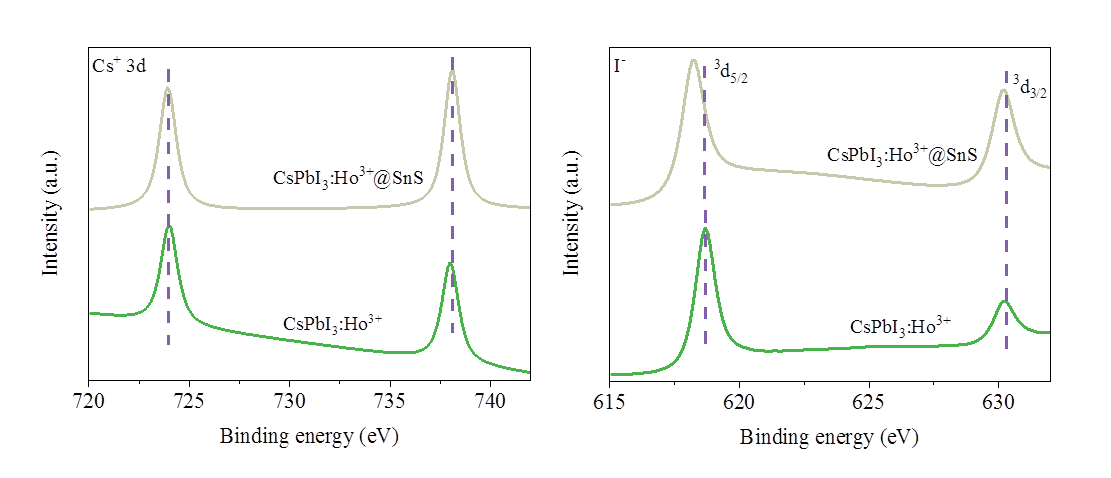


Figure S8. XPS spectra of Cs 3*d* and I 3*d* in CsPbI_3_:Ho^3+^ PQDs and CsPbI_3_:Ho^3+^@SnS heterostructures QDs.


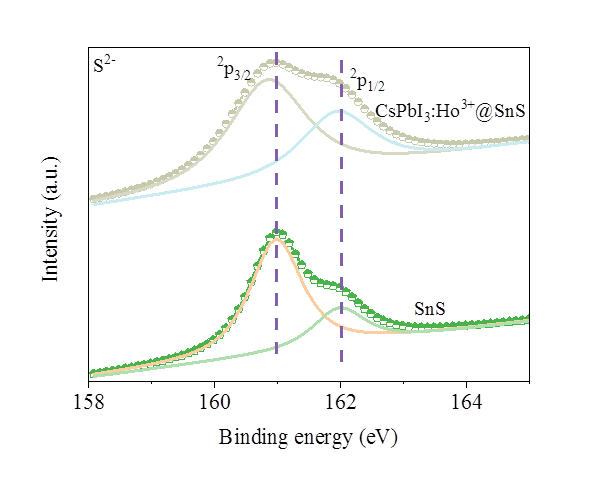


Figure S9. XPS spectra of S 2*p* in SnS QDs and CsPbI_3_:Ho^3+^@SnS heterostructures QDs.


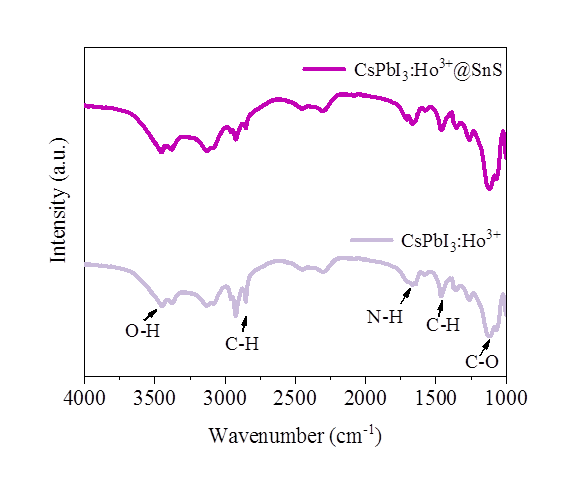


Figure S10. FTIR spectra of CsPbI_3_:Ho^3+^ PQDs and CsPbI_3_:Ho^3+^@SnS heterostructured QDs.


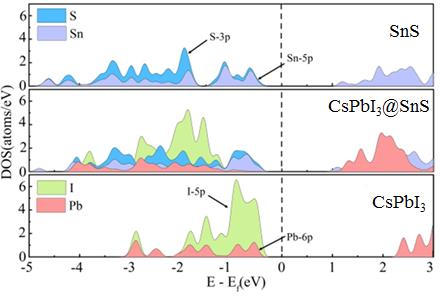


Figure S11. Calculated density of states (DOS) of the SnS, CsPbI_3_:Ho^3+^@SnS and CsPbI_3_:Ho^3+^.


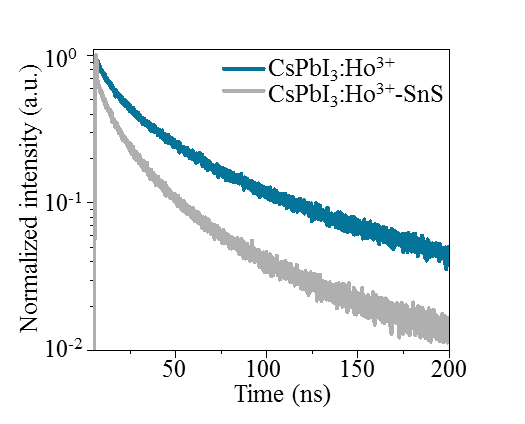


Figure S12. PL lifetime of CsPbI_3_:Ho^3+^ and CsPbI_3_:Ho^3+^@SnS heterostructures QDs.


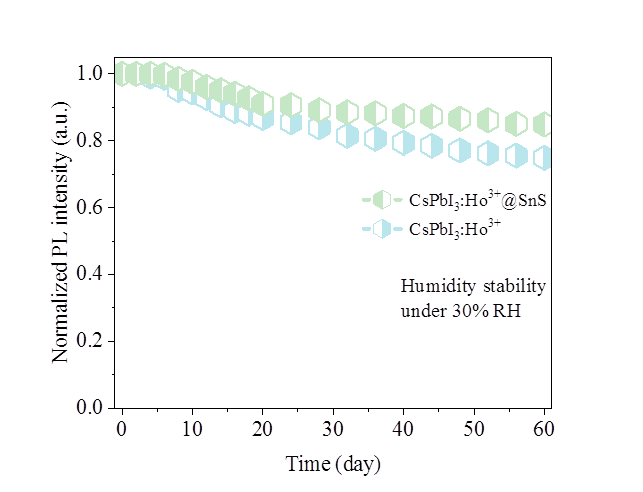


Figure S13. Normalized PL intensity of CsPbI_3_:Ho^3+^ PQDs and CsPbI_3_:Ho^3+^@SnS QDs as a function of storage time.


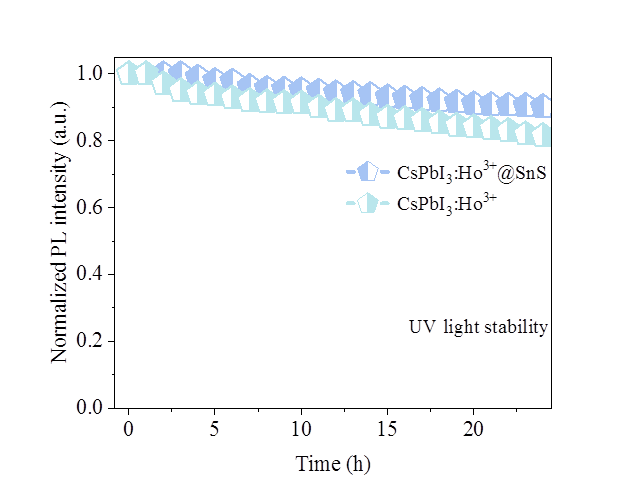


Figure S14. Normalized PL intensity of CsPbI_3_:Ho^3+^ PQDs and CsPbI_3_:Ho^3+^@SnS QDs as a function of UV light.


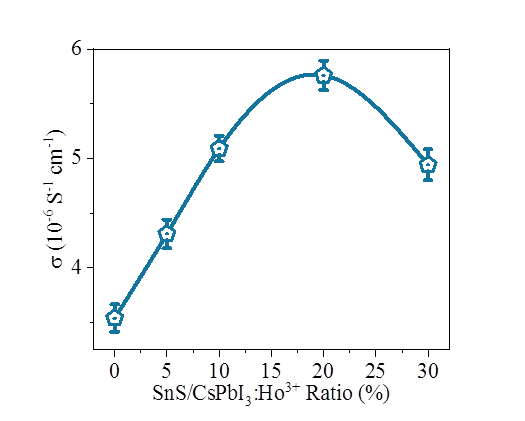


Figure S15. The conductivity of CsPbI_3_:Ho^3+^@SnS QDs heterojunctions with different SnS QDs concentration.


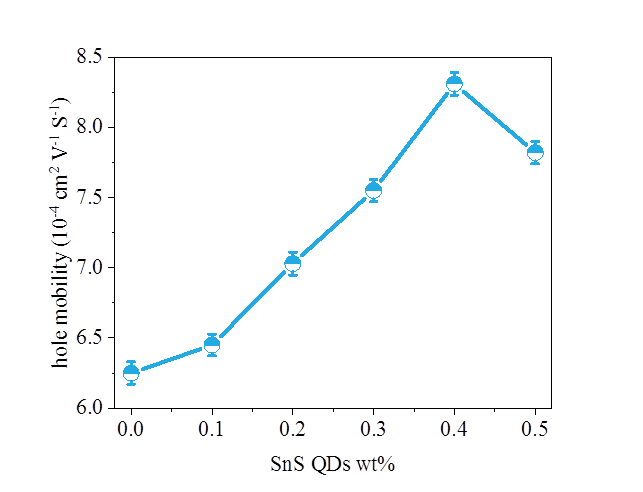


Figure S16. The hole mobility of SnS QDs doped into Spiro with different concentrations.


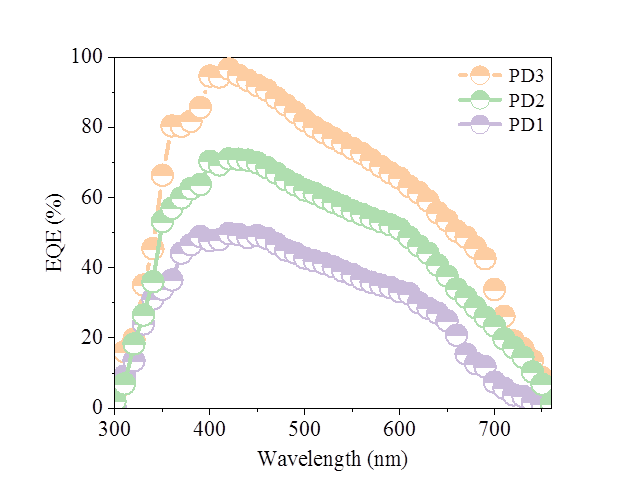


Figure S17. EQE of PD1-PD3.


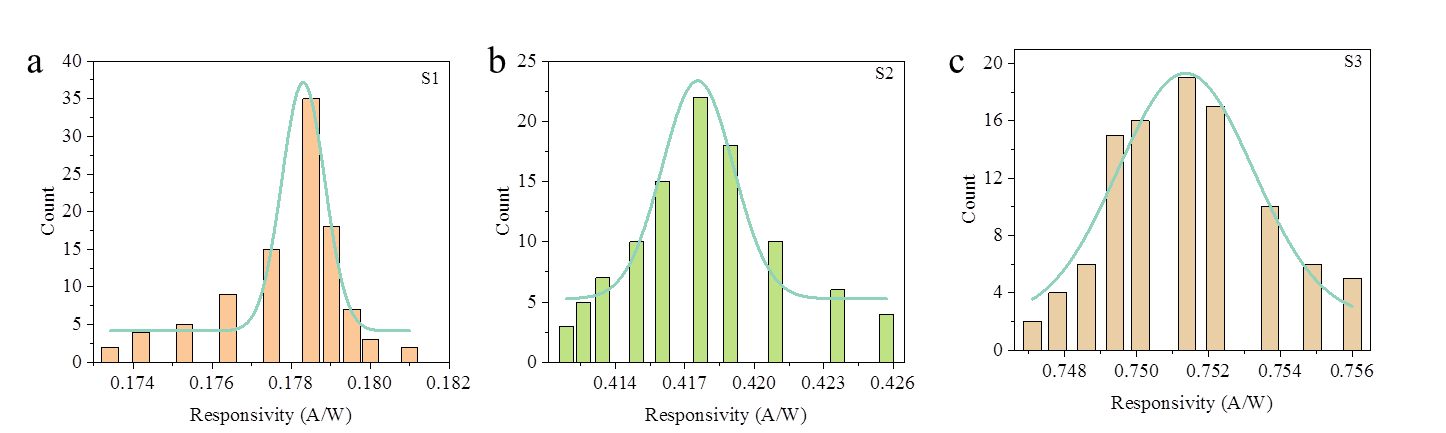


Figure S18. Histogram of R of the PD1-PD3.


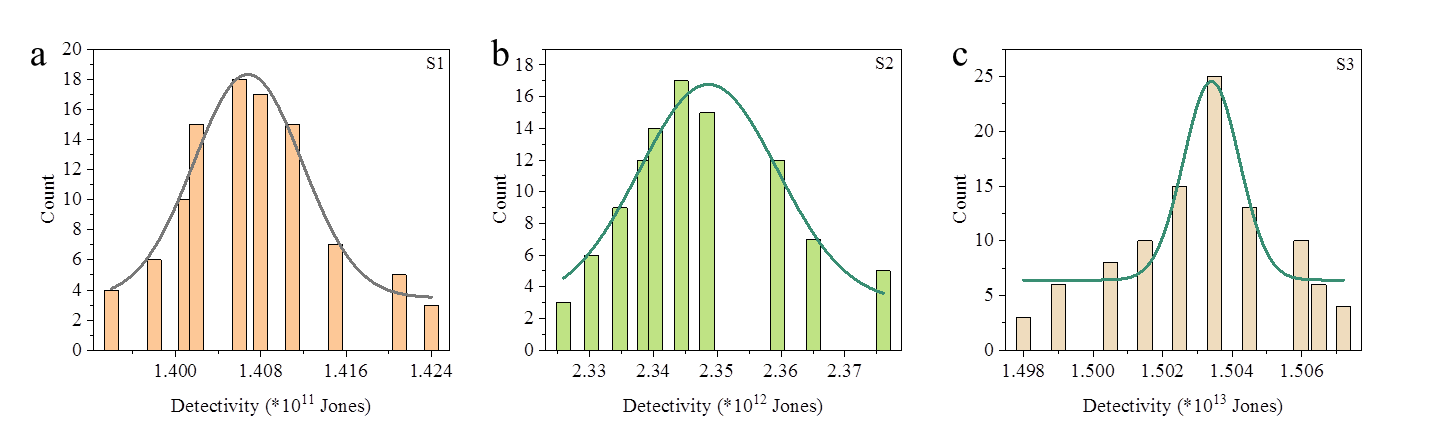


Figure S19. Histogram of D* of the PD1-PD3.


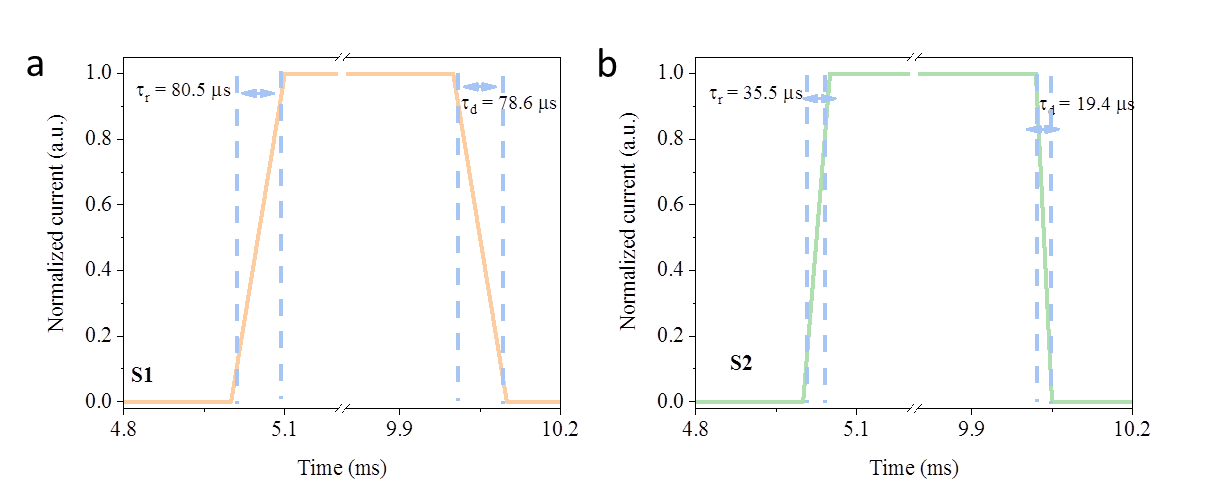


Figure S20.The rise and decay times of PD1 (a) and PD2 (b).


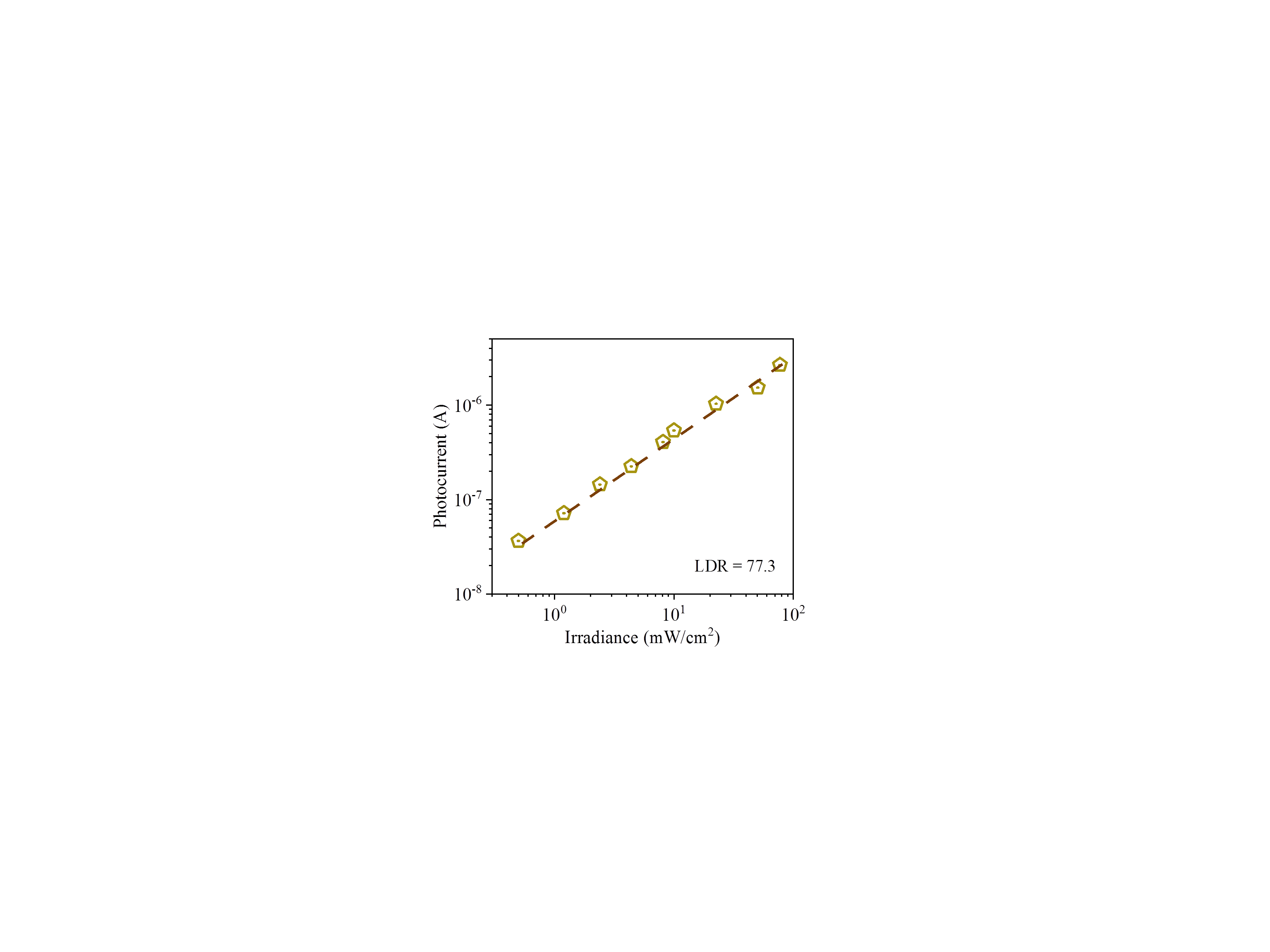


Figure 21. Photocurrent of the devices under different irradiation powers ranging.


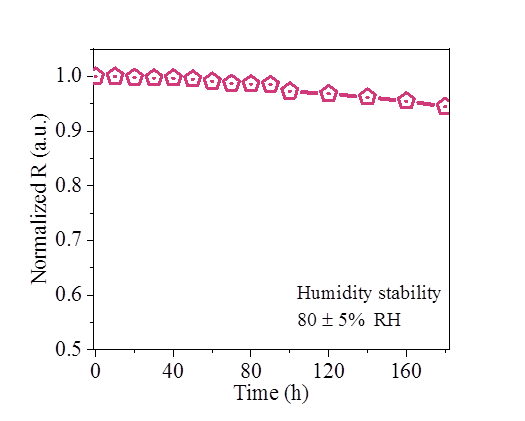


Figure 22. Humidity stability of the PDs.


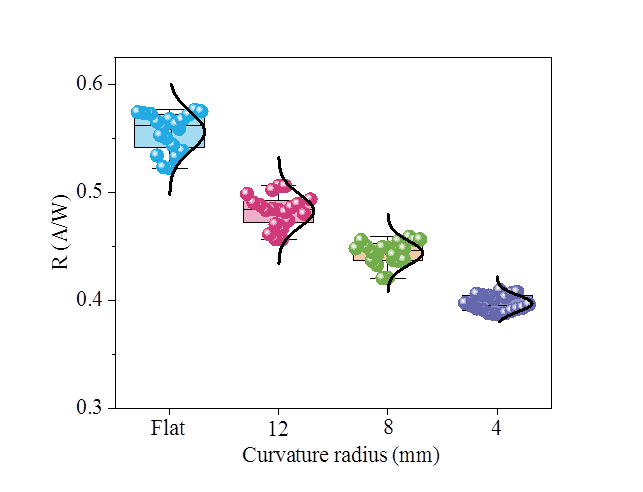


Figure S23. The R of the flexible PDs under different curvature radius.


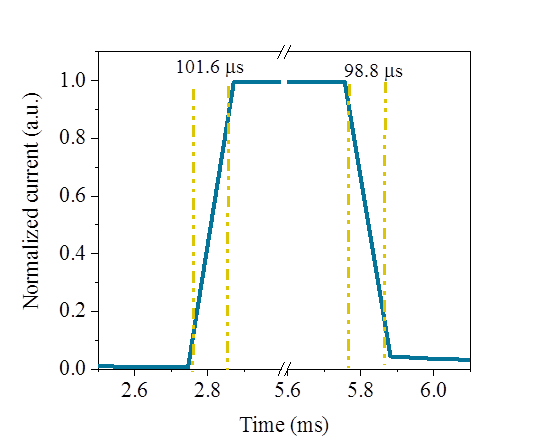


Figure S24. The response time of the flexible PDs.


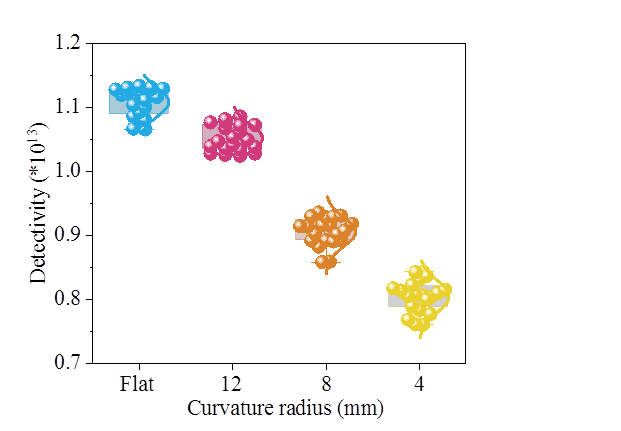


Figure S25. The detectivity of the flexible PDs under different curvature radius.


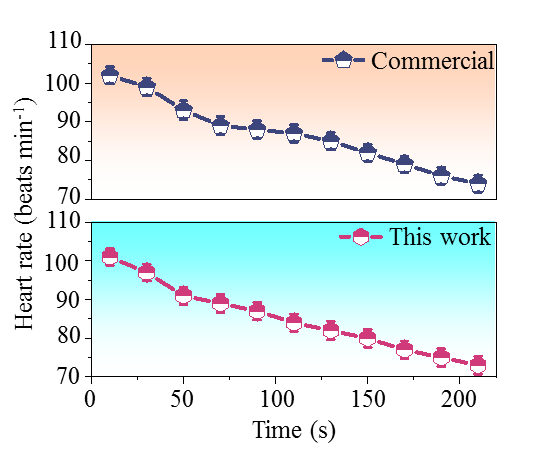


Figure S26. Comparison of this work and the commercially available pulse sensor after exercise.


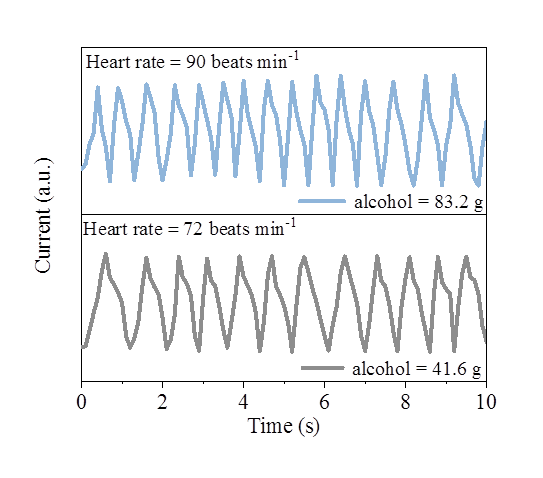


Figure S27. Photocurrent response with different alcohol intake by our device.


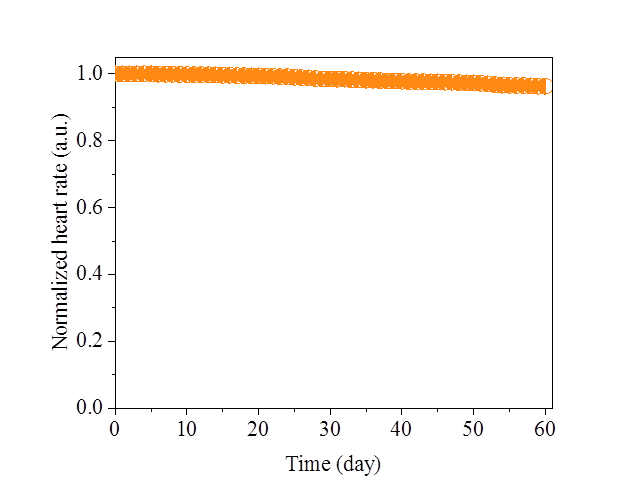


Figure S28. The long term- stability of the device.
